# Context-dependent social evaluation in 4.5-month-old human infants: the role of domain-general versus domain-specific processes in the development of social evaluation

**DOI:** 10.3389/fpsyg.2014.00614

**Published:** 2014-06-18

**Authors:** J. K. Hamlin

**Affiliations:** Department of Psychology, University of British ColumbiaVancouver, BC, Canada

**Keywords:** social evaluation, infancy, cooperation, domain-general processes, domain specificity, context-dependence

## Abstract

The ability to distinguish friends from foes allows humans to engage in mutually beneficial cooperative acts while avoiding the costs associated with cooperating with the wrong individuals. One way to do so effectively is to observe how unknown individuals behave toward third parties, and to selectively cooperate with those who help others while avoiding those who harm others. Recent research suggests that a preference for prosocial over antisocial individuals emerges by the time that infants are 3 months of age, and by 8 months, but not before, infants evaluate others’ actions in context: they prefer those who harm, rather than help, individuals who have previously harmed others. Currently there are at least two reasons for younger infants’ failure to show context-dependent social evaluations. First, this failure may reflect fundamental change in infants’ social evaluation system over the first year of life, in which infants first prefer helpers in any situation and only later evaluate prosocial and antisocial actors in context. On the other hand, it is possible that this developmental change actually reflects domain-general limitations of younger infants, such as limited memory and processing capacities. To distinguish between these possibilities, 4.5-month-olds in the current studies were habituated, rather than familiarized as in previous work, to one individual helping and another harming a third party, greatly increasing infants’ exposure to the characters’ actions. Following habituation, 4.5-month-olds displayed context-dependent social preferences, selectively reaching for helpers of prosocial and hinderers of antisocial others. Such results suggest that younger infants’ failure to display global social evaluation in previous work reflected domain-general rather than domain-specific limitations.

## INTRODUCTION

Human cooperation presents an evolutionary puzzle. Although human beings are easily the most cooperative and altruistic species on earth (Tomasello, 2009; Melis and Semmann, 2010), helping others is personally costly and there is uncertainty that such efforts will be returned. Thus, cooperative systems are constantly in danger of being overtaken by individuals who reap the benefits of others’ costly prosocial acts but do not take costs to help others in return. To solve the puzzle of how cooperation could have evolved, theorists argue that human prosocial motivations must emerge *in tandem with* capacities for social evaluation and partner choice. That is, cooperation is possible because humans are selective cooperators: they readily assess others’ cooperative potential and choose social partners accordingly, allowing them to pay the costs of cooperating only to those likely to pay them back. Non-cooperators, on the other hand, are shunned or actively punished, making non-cooperation a less beneficial strategy overall (e.g., Trivers, 1971; Axelrod, 1984; Alexander, 1987; Cosmides, 1989; Boyd and Richerson, 1992; Nowak and Sigmund, 1998; Price et al., 2002; Panchanathan and Boyd, 2003). Although some claim that humans evolved capacities to detect cheaters in social exchanges specifically (e.g., Delton et al., 2012), others treat sociomoral evaluation and partner choice as more general solutions to various problems inherent to group living; promoting bigger and bigger acts of altruism, curbing aggression between group members, allowing for the establishment of a variety of group norms, etc. (e.g., Alexander, 1987; Boyd and Richerson, 1992; Sober and Wilson, 1998; Flack and deWaal, 2000; Katz, 2000; Hammerstein, 2003; Hardy and Van Vugt, 2006; Barclay and Willer, 2007; Nesse, 2007; Boehm, 2012).

Supporting the possibility that humans developed capacities for social evaluation and partner choice along with tendencies toward cooperation and prosociality comes from recent evidence that very young infants engage in third party social evaluations, suggesting they are not solely the result of socialization and learning processes (reviewed in Hamlin, 2013a). Specifically, as early as 3 months of age infants prefer puppet characters who help, versus prevent, third parties in achieving their unfulfilled goals, despite having no immediate “stake” in the interaction and not knowing anyone involved. Infants’ preferences for prosocial versus antisocial puppets are measured by selective attention in 3-month-olds (who cannot yet reach) and by both selective looking and reaching in older infants, and occur in response to helpers and hinderers of several different goal scenarios, including a goal to reach a particular location, to have a dropped object returned, and to obtain an object that is beyond a physical barrier (Hamlin et al., 2007, 2010, 2013b; Hamlin and Wynn, 2011). Critically, infants do not distinguish characters who direct identical physical actions toward an inanimate object or toward an agent who was not clearly demonstrating an unfulfilled goal, suggesting their preferences do not reflect liking or disliking particular lower-level perceptual aspects of the events (Hamlin, in revision; Hamlin et al., 2010; Hamlin and Wynn, 2011; c.f. Scarf et al., 2012a and response by Hamlin et al., 2012a). Finally, by 8–10 months of age infants’ evaluations are based on others’ *intentions* to help or hinder rather than whatever outcomes happened to occur: infants prefer those who try but fail to help over those who try but fail to hinder, but they do not distinguish those who actually helped and hindered if they did not know they were doing so (e.g., Hamlin, 2013b; Hamlin et al., 2013b).

Of course, adults’ social evaluations are not limited to simple heuristics whereby all “locally” intentional prosocial acts are good and all antisocial ones are bad (Heider, 1958). Instead, adults demonstrate more “global” evaluations, readily assessing the very same behaviors differently in different contexts. For example, even though punishment is itself antisocial, adults readily punish those who have behaved antisocially and approve of others who do so (see Bright and Keenan, 1995; Maurer, 1999; Barclay, 2006; Gürerk et al., 2006; Friedland, 2012), and like those who share their social tastes and distastes, even when shared distaste is signaled by an antisocial act (as illustrated by the phrase “the enemy of my enemy is my friend,” e.g., Heider, 1958; Aronson and Cope, 1968; Gawronski et al., 2005). In a study exploring one type of context-dependent social evaluation in infancy, Hamlin et al. (2011; see also Hamlin et al., 2013a) compared infants’ preferences for Givers versus Takers of a dropped ball when the individual who dropped it had either just helped or just hindered an unknown third party in his goal to open a box. Specifically, we hypothesized that if infants engage in only local evaluations they should prefer Givers to Takers across the board; if infants are capable of global evaluations their preferences should differ based on the past behavior of the targeted individual. Both 8-month-olds infants and 19-month-old toddlers showed markedly different choice patterns depending on the target of giving and taking, selecting givers when targets were prosocial and takers when targets were antisocial. To address whether infants’ context-specific preferences reflect mere “valence-matching,” or a preference for those whose interactions maintain the same valence over time, additional groups of 8- and 19-month-olds chose between givers and takers when a target had previously *received*, rather than *performed*, an antisocial act. Victims of antisocial behaviors do not deserve further mistreatment, nor do adults wish to befriend their enemies, but they are clearly (however unwilling) participants in a negatively valenced act, and continuously struggle and fail to achieve a goal (see Skerry and Spelke, 2014, for evidence that infants appreciate the emotional consequences of goal achievement and failure by 8 months of age). If infants simply prefer valence-matchers without analyzing who did what to whom or distinguishing between various forms of negative valence present during hindering, then they should be even *more* likely to choose takers from victims than from hinderers. Critically, both 8- and 19-month-olds preferred givers to victims, ruling out the low-level valence-matching alternative for infants’ context-specific choices (but see Scarf et al., 2012b, and response by Hamlin et al., 2012b).

Five-month-olds in Hamlin et al. (2011) were tested on the very same procedures but showed no evidence of context-dependence (nor, notably, of valence-matching): they preferred those who gave to versus took from all targets, whether prosocial or antisocial. This performance difference suggests that the ability to demonstrate global social evaluations develops between 5 and 8 months after birth. That said, the nature of this development remains unclear. On the one hand, development between 5 and 8 months may occur within the domain of social evaluation itself. Infants might first possess relatively simple “helpful = good and/or harmful = bad” heuristics that are impervious to contextual information of any kind, and later develop the ability to evaluate prosocial and antisocial actions in context. Such domain-specific change could be prompted by infants’ everyday experiences: as infants age and become increasingly mobile they are presumably confronted with more and more locally antisocial behavior performed by individuals infants are sure they like (their caregivers) toward individuals infants are sure they like (themselves, their peers and/or siblings). These experiences might then drive infants to adjust their rigid social evaluation system in order to incorporate information related to who did what to whom and why. That is, in a process of accommodation (e.g., Piaget, 1928), global social evaluation might emerge as infants encounter, and are motivated to make sense of, apparent inconsistencies in their increasingly complex social world. Notably, 8 months is also the time when mentalistic third party social evaluation has first been observed in infants (Hamlin, 2013b).

A second (non-mutually-exclusive) possibility for younger infants’ failure is that 5-month-olds are limited in terms of memory, processing speed/capacity or other domain-general ability relative to 8-month-olds. Indeed, the methodology used in Hamlin et al. (2011) was extremely complex relative to past work on social evaluation in younger infants, and may have placed insurmountable demands on 5-month-olds’ processing and memory capacities. To illustrate, infants in Hamlin et al. (2011) saw two different types of prosocial and antisocial interactions within the same study, both the box and the ball scenarios. Although infants readily distinguish prosocial from antisocial others when shown either one of these scenarios, no previous work has demonstrated they can do so when shown both types, much less integrate information across the two. In addition, while in past studies infants have had to keep track of three unique characters who are all onstage together at the start of each event, in the global evaluation procedure infants must keep track of five distinct characters, only three of whom are ever onstage at once. Finally, infants in Hamlin et al. (2011) were not only given more information to process than in past work, they also had less time to process it: past work has utilized a habituation procedure in which infants are shown prosocial and antisocial events repeatedly until a pre-specified criterion is reached (between three and seven events each; habituation is taken to indicate sufficient event processing, for review see Colombo and Mitchell, 2009), whereas infants in Hamlin et al. (2011) saw just one prosocial and one antisocial event in each of the box and ball scenarios. Therefore, perhaps 5-month-olds selected givers over takers following the ball scenario simply because they initially *failed to process* or subsequently *forgot* what the target in the ball shows had done, and so they evaluated givers and takers as if the target was an unknown third party. If so, then the procedure was not actually a test of 5-month-olds’ capacity for context-dependent social evaluation in the first place.

Consistent with this possibility, there are clear improvements in infants’ processing and memory capacities with age. Younger infants are slower to process information than are older infants, younger infants forget information faster after equivalent exposure than do older infants, and younger infants show striking difficulty retrieving information over changes in context whereas older infants do better (for reviews see Roveé-Collier, 1997, 1999; Hayne, 2004; Bauer, 2007; Colombo and Mitchell, 2009). Neuroimaging work has linked functional development in learning and memory in infancy to changes in temporal cortical memory networks known to underlie declarative memory in adults, with significant changes happening during the second half-year of life (reviewed in Richmond and Nelson, 2007). Together, this work suggests that given equal exposure time 8-month-olds should be, on average, markedly better than 5-month-olds at encoding, retaining, and retrieving information from one phase of an experiment to the next.

## MOTIVATION FOR THE CURRENT STUDIES

The current studies were designed to distinguish between domain-specific and domain-general accounts of the observed difference in 5- and 8-month-olds’ social evaluations in Hamlin et al. (2011). Infants from 3.5 to 5.5 months of age were tested, with an average age of 4.5 months. All methodologies were identical to Hamlin et al. (2011), except that memory and processing demands were reduced: rather than being shown one prosocial and one antisocial box event in the first phase of the procedure, infants were habituated to prosocial and antisocial box events, seeing alternating events repeatedly until their attention following each event decreased by half (details below). Dominant theoretical approaches to habituation characterize it as a process of alignment, by which an internal representation of an external stimulus becomes more similar to the stimulus itself (e.g., Sokolov, 1963; see review in Colombo and Mitchell, 2009). Therefore, habituating infants to box events should in some way or another sharpen their internal representations of the would-be Targets of giving and taking, which they might utilize while observing giving and taking. After habituation, 4.5-month-olds were shown just one giving and one taking ball event before choosing between the giving and taking puppets, as in Hamlin et al. (2011).

If 4.5-month-olds in the current study perform as 5-month-olds in Hamlin et al. (2011), consistently choosing givers over takers even after being habituated to prosocial and antisocial box events, this would lend support the possibility that differences in social evaluation at 5 and 8 months reflect some change in the system of social evaluation itself, whereby infants move from initially rigidly viewing helping as good and hindering as bad to incorporating contextual nuance into their social assessments. On the other hand, if 4.5-month-olds choose Givers to Prosocial Targets but Takers from Antisocial Targets, it would suggest that younger infants’ failure to demonstrate global social evaluation in Hamlin et al. (2011) was due to difficult task demands combined with domain-general limitations in memory and processing capacities.

## EXPERIMENT 1: PROSOCIAL AND ANTISOCIAL TARGETS

### METHOD

#### Participants

Fifty-five full-term and typically developing infants between 3.5 and 5.5 months of age participated. An additional 22 infants began or completed the procedure but were not included in the final sample due to fussiness (13 infants), procedural error (4), failure to choose either puppet (4), or parental interference (1). Data collection ended somewhat early (the original intention was 32 infants/condition) in time to submit the manuscript for this special issue; in total there were 28 infants in the Prosocial Target condition (14 females; mean age = 4 months; 19 days; range = 3;16–5;16) and 27 infants in the Antisocial Target condition (16 females; mean age = 4 months; 17 days; range = 3;18–5;16). Twenty of 28 infants in the Prosocial Target condition and 19 of 27 in the Antisocial Target condition had were first born and had no siblings at the time of testing.

#### Procedures

All procedures were approved by the Behavioral Research Ethics Board at the University of British Columbia and conform to relevant regulatory standards.

**FIGURE 1:**
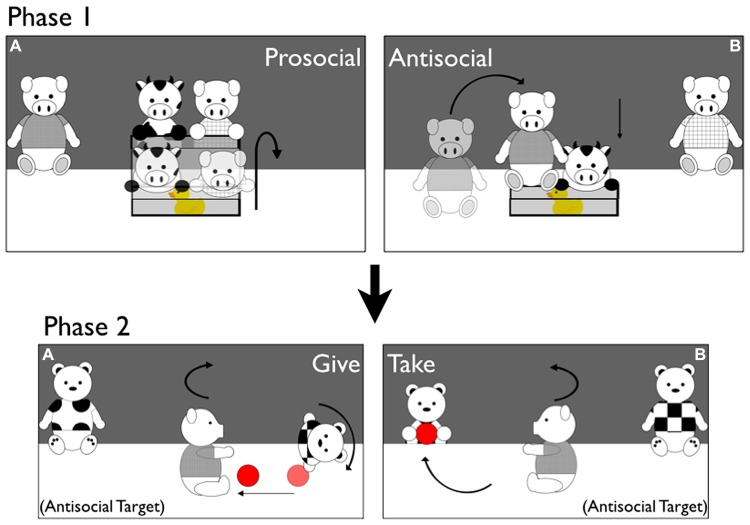
**Stimuli**. Depictions of Prosocial/Antisocial puppet shows during Phase 1 and Giving/Taking puppet shows during Phase 2 of Experiment 1 (example of the Antisocial Target condition). Phase 1 **(A)** Prosocial Box Events. The Cow enters the stage and tries but fails to open the box. The Prosocial Pig helps him open it. The Cow lies down on top of the toy inside the box, and the Prosocial Pig runs offstage. Phase 1 **(B)** Antisocial Box Events. The Cow enters the stage and tries but fails to open the box. The Antisocial Pig jumps on top of the box, slamming it shut. The Cow lies down next to the box without the toy, and the Antisocial Pig runs offstage. Phase 2 **(A)** Giving Ball Events. The Antisocial Pig from Phase 1 enters the stage, and picks up a ball resting in the center. The Pig drops and catches the ball several times, and then drops the ball toward the Giver Tiger. The Pig turns to “ask” for the ball back, and the Giver rolls the ball back to him and runs offstage. Phase 2 **(B)**: taking Ball Events. The Antisocial Pig enters and plays with the ball as in Giving Events. He then drops the ball toward the Taker Tiger, and asks for it back as in Giving Events. The Taker Tiger runs offstage, stealing the ball away.

Stimuli and procedures are identical to Hamlin et al. (2011) unless otherwise noted, and are depicted in **Figure 1**. Infants participated in two Stimuli Phases and a Choice Phase. For each Stimuli Phase, infants sat in their parent’s lap before a table (*W*: 122 cm) surrounded on three sides with blue curtains; a curtain with cartoon animal cutouts on it (85 cm from the infants) could be lowered to occlude the puppet stage so stimuli could be reset between events. Parents were instructed to sit quietly with their infants and not attempt to influence them in any way. Before the start of the study parents practiced getting into the appropriate position for the Choice Phase, turning 90° to the right and moving back about 30 cm (placing their feet on a duct tape line on the floor), perching their infants at the front of their knees (not leaning back against their chest), and holding them tightly around the lower abdomen. Parents were told how important it is that infants face straight ahead and have sufficient trunk support to ensure clear reaches at this young age. Infants were *habituated to* up to 14 puppet events in Stimuli Phase 1, and were *familiarized to* exactly two puppet events in Stimuli Phase 2. Additional details of each Phase are described below.

***Stimuli Phase 1: box helping and hindering events.*** Depicted in **Figures 1A,B**. The curtain rose to reveal two pink pigs (one in a blue shirt, one in green) resting at the back corners of the puppet stage; a clear box containing a brightly-colored toy rested at the center of the stage, approximately 20 cm in front of the pigs. To begin each and every event, a Cow puppet wearing a yellow t-shirt entered from the back center of the stage, and ran around one side of the box and “looked” inside twice, as if seeing the toy inside. The Cow then jumped up on top of the nearest corner of the box lid, and lifted the box lid a small amount a total of five times, lowering it in between as though unable to open the box. During *Prosocial Events*, during the fifth struggle the Prosocial Pig (resting in the corner of the opposite side of the stage from where the Cow was struggling) ran forward, grasped the opposite corner of slightly open box lid, and opened the box together with the Cow. The Cow jumped into the open box, lay his body down on top of the toy inside, and paused. The Prosocial Pig then jumped off the box lid and ran offstage to complete the event. During *Antisocial Events*, during the fifth struggle the Antisocial Pig ran forward (the side of the box the Cow struggled with alternated per event so that the Prosocial and Antisocial Pigs could remain in the same corners throughout the procedure) and jumped on top of the slightly-open box lid, slamming it shut. The Cow jumped off the box, lay his body down on the stage, and paused, and the Antisocial Pig jumped off the box and ran offstage.

Once the Prosocial/Antisocial Pig ran offstage at the end of each event, an online coder coded infants’ attention toward and away from the puppet stage using a key-press via the program *jHab* (Casstevens, 2007). Coding ended when infants looked away from the stage for 2 consecutive seconds or after 30 total seconds elapsed, as indicated by a “ding” from the *jHab* program. After each ding the curtain was lowered and the next event was readied. Infants viewed prosocial and antisocial events in alternation until they reached a pre-set habituation criterion in which their attention over three consecutive events summed to less than half their attention over the first three events that themselves summed to 12 s or more. If infants failed to reach this criterion, they were shown 14 total events in Stimuli Phase 1. In Phase 1, the event order, side of stage, and t-shirt color of the Prosocial Target was counterbalanced.

Once infants completed Phase 1, the online coder and puppeteer from Phase 1 switched places. The new puppeteer (former coder) did not know which puppet had performed which action during Phase 1, and remained blind to condition while puppeteering Phase 2 by reading the shirt color of the Target Pig for Phase 2 from a script only s/he had access to. The new coder, despite having puppeteered during Phase 1 and knowing which Pig was which, could not see the stage during Phase 2 and so did not know which Pig was the Target of Giving and Taking.

***Stimuli Phase 2: ball giving and taking events.*** The curtain rose to reveal two Tiger puppets, wearing a pink and a purple t-shirt, resting at the back corners of the stage. A ball rested at the center of the stage. Depending on condition, either the Prosocial Pig from Phase 1 (in the Prosocial Target condition) or the Antisocial Pig from Phase 1 (in the Antisocial Target condition) entered from behind the back curtain, and ran forward to grasp the ball. The Target then bounced twice, holding the ball, and then released and grasped the ball, as though playing with it. The Target repeated this jump-release-retrieve sequence twice more; on the fourth release the ball rolled toward one side of the stage or the other. During *Giving Events*, the Giver (closest to the ball) ran forward and grabbed the ball. The Target then turned toward the Giver and opened its arms wide, as though “asking” for the ball back; the Giver turned toward the Target as though acknowledging him, and both puppets turned back to face the infant simultaneously. This sequence repeated once more; the third time the Target turned toward the Giver, the Giver rolled the ball back to the Target (a distance of approximately 30 cm), and then ran offstage. The Target turned back to face the infant, holding the ball, and all action paused. During *Taking Events*, the Taker (closest to the ball because it dropped toward the other side of the stage) ran forward and grabbed the ball. The Target “asked” for its ball back twice as in Giving Events; on the third request the Taker rushed offstage, stealing the ball away. The Taker turned back to face the infant without the ball and all action paused. Infants’ attention to each event was recorded as in Phase 1. Unlike in Phase 1, infants in Phase 2 were shown a total of two events, one Giving and one Taking (as in Hamlin et al., 2011). During Stimuli Phase 2, the t-shirt color, event order, and side of the Giver and Taker were counterbalanced in each condition.

After Stimuli Phase 2, parents were instructed to get into position for choice, and were asked to adjust their infants if necessary. Once infants were in the appropriate position, parents were asked to close their eyes.

***Choice.*** The coder from Phase 2, who knew neither which Tiger was the Giver or the Taker nor whether each infant was in the Prosocial or the Antisocial Target condition, presented the choice. The puppeteer from Phase 2 placed puppets in the choice presenters’ appropriate hands by reading from a script only s/he had access to, and the choice presenter hid the Tigers behind her back as she appeared from behind the curtain that had been on the infants’ right during the puppet shows (now about 45° to infants’ left). The choice presenter kneeled directly in front of the infant, said “Hi!” and established eye contact. S/he then brought both puppets into view (but out of reach, approximately 60 cm away) as she said said “Look!”. Infants were required to look toward each puppet; if an infant failed look at both spontaneously when they were first introduced, the presenter would shake one or both puppets as necessary to ensure the infant saw each one (with instructions that infants’ gaze should land on each puppet for as brief a time a possible). Finally, the choice presenter said “Hi!” again, reestablished eye contact so that an infant did not simply choose whichever puppet s/he had just been looking toward, and moved the Tigers within reach (approximately 15–30 cm away), saying “Who do you like?”. Each infant’s “choice” was identified online by the choice presenter as the first puppet contacted via a visually guided reach (touching a puppet preceded immediately by looking at it). The side of the Giver/Taker was counterbalanced during choice. An additional 25% of infants’ choices in each condition were recoded for reliability purposes; reliability was 100%.

### RESULTS

Attention was analyzed using *t*-tests and ANOVAs; statistics reported include 95% Confidence Intervals (CIs). Choices were analyzed using non-parametric tests for categorical data (binomial tests for comparing a given choice distribution to chance (50%); Fisher’s Exact Tests and Chi-squares for comparing choice distributions across conditions) and also include 95% CIs. All statistics were generated via SPSS, www.vassarstats.net (for non-parametric analyses) and ESCI (Cumming, 2012).

#### Attention during Stimuli Phase 1

***Rate of habituation.*** Across conditions, infants habituated in an average of 8.73 events (SEM = 0.37). This number differed marginally by condition (variance assumption violated, independent-samples *t*(49) = -1.90, *p* = 0.065, Cohen’s *d* = 0.51, 95% CI of difference [-2.78,.08]). Infants in the Prosocial target condition habituated in an average of 9.39 (SEM = 0.58; 95% CI [8.20, 10.58]) events (22/28 infants habituated within 14 events) and infants in the Antisocial Target condition habituating in 8.04 (SEM = 0.42, 95% CI [7.18, 8.90]) events (26/27 infants habituated within 14 events). The difference in the percentage of infants per condition who habituated within 14 events also approaches marginal significance (2X2 Fisher’s Exact Test, *p* = 0.10; 95% CI on the difference [-1, 36]). As infants in both conditions viewed exactly the same events during Phase 1, and because during Phase 1 both puppeteers and coders were blind to infants’ condition, these marginal interactions are considered spurious (in addition, there were no effects of whether infants reached habituation during Phase 1 on infants’ puppet choices; see below).

***Attention to prosocial versus antisocial events.*** Infants attended equally to Prosocial and Antisocial Events across conditions, whether comparing looks to the first instance of each [first Prosocial (SEM) = 11.27 s (1.18); first Antisocial (SEM) = 10.17 s (1.07); paired-*t*(54) = 0.90, *p* = 0.37, *d* = 0.13, 95% CI [-5.64, 3.44] or to the average across the first 3 instances of each [as per the habituation criterion, all infants saw at least three of each event type; average first three Prosocial (SEM) = 8.78 s (0.80); average first three Antisocial (SEM) = 8.18 s (0.72); paired *t*_54_ = 0.90, *p* = 0.37, *d* = 0.11, 95% CI [-3.68, 2.46]. As expected given that all infants viewed the exact same events during Phase 1, repeated-measures ANOVAs with condition as a between-subjects factor revealed that infants’ relative attention to Prosocial versus Antisocial Events did not differ by condition (first Prosocial/Antisocial: *F*_1,53_ = 0.67, *p* = 0.41, $\eta_{p}^{2}$ = 0.01; average first three Prosocial/Antisocial: *F*_1,53_ = 0.56, *p* = 0.46, $\eta_{p}^{2}$ = 0.01). This lack of attention difference to Prosocial versus Antisocial acts suggests that 4.5-month-old infants do not hold baseline expectations for whether unknown third parties will help or hinder other unknown third parties.

#### Attention to giver and taker events during Phase 2

Across conditions, infants attended equally to Giver and the Taker Events [Giver (SEM) = 6.24 (0.78), Taker (SEM) = 7.32 (0.87); paired-*t*(54) = –1.26, *p* = 0.22, *d* = 0.17, 95% CI [-2.25, 4.39]; this did not differ by condition (repeated-measures ANOVA, *F*_1,53_ = 0.02, *p* = 0.90, $\eta_{p}^{2}$ = 0.00). These results replicate what was reported in Hamlin et al. (2011) and suggest that 4.5-month-olds do not hold expectations for how independent third parties will treat those they (the infants) know have helped or hindered others (see Meristo and Surian, 2013 for positive evidence with older infants and fair/unfair distributors).

#### Choice

**FIGURE 2:**
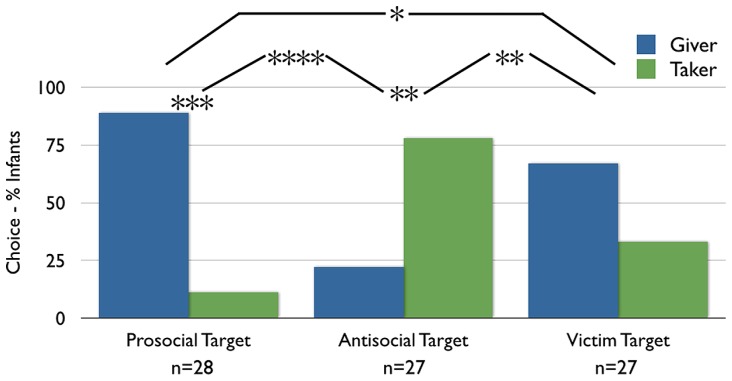
**Results**. Infants’ choices for the giver versus the taker (in %) across conditions. *p < 0.05, **p < 0.01, ***p < 0.00005, ****p < 0.0000005.

***Preliminary analyses.*** Choice results are depicted in **Figure 2**. When collapsed across conditions, preliminary binomial tests revealed no effects of side, color, or giving/taking action on infants’ choices (*p*’s > 0.41). Both across and within conditions, habituators and non-habituators chose in the direction of the hypothesis at equal rates (Fisher’s Exact *p*_Across_ = 0.59, *p*_WithinProsocial_ = 1.0, *p*_WithinAntisocial_ = 1.0). Interestingly, males in the Antisocial Target condition were more likely to choose the Taker than were females (Fisher’s Exact *p* = 0.05); this difference was not observed in the Prosocial Target condition (*p* = 1.0) nor collapsed across both (*p* = 0.16) and so is not addressed further. The choice pattern of infants with siblings did not differ from those without (Fisher’s Exact *p*’s > 0.54). Finally, a multivariate ANOVA on whether infants chose with or against the hypothesis and whether infants chose the Giver or Taker with age as a covariate revealed no effects of age on infants’ choices (*F*’s_1,54_ < 1.05, *p*’s > 0.31, $\eta_{p}^{2}$ ’s < 0.03).

***Choice of givers versus takers.*** Infants’ preference for the Giver versus the Taker puppet differed significantly by condition [Pearson’s χ^2^ (*df* = 1) = 25.14, *p* < 0.0001]. Specifically, infants were 67% more likely to choose the Giver in the Prosocial Target than in the Antisocial Target condition (95% CI on difference does not cross 0 [42, 81]). Infants in the Prosocial Target condition significantly preferred the Giver over the Taker (25 of 28 infants; binomial *p* = 0.00003; 95% CI on the percentage choosing the Giver is entirely above chance (50%) [73, 96]), whereas infants in the Antisocial Target condition significantly preferred the Taker over the Giver (21 of 27 infants; binomial *p* = 0.006; 95% CI on percentage choosing the Giver is entirely below chance [10, 41]). Infants’ likelihood to choose in the direction of the hypothesis did not differ by condition (Fisher’s Exact Test, *p* = 0.30; 95% CI [-31, 9]): infants were equally likely to choose Givers to Prosocial Targets as they were to choose Takers to Antisocial Targets. Finally, infants’ rate of choosing Givers versus Takers differed significantly between the current Antisocial Target condition, in which infants were habituated during Phase 1 (21 of 27 chose Taker), and the Antisocial Target condition of Hamlin et al. (2011), in which infants were only familiarized during Phase 1 [2 of 16 chose Taker; Pearson’s χ^2^ (*df* = 1) = 17.21, *p* < 0.0001], reflecting a 65% [35, 80] difference in rate of choosing the Taker.

### DISCUSSION

Results from Experiment 1 suggest that given more time to process the initial prosocial and antisocial acts of the eventual targets of giving and taking, even 4.5-month-olds demonstrate context-dependent social evaluations, preferring those who are nice (over mean) to nice puppets and those who are mean (over nice) to mean puppets. To rule out simple valence-matching effects for infants’ choices, a new group of 4.5-month-olds chose between a Giver to and a Taker from a Victim Target as in Hamlin et al. (2011). It was predicted that 4.5-month-olds would prefer the Giver to the Taker in the Victim Target condition, as had both 8- and 19-month-olds in Hamlin et al. (2011).

## EXPERIMENT 2: VICTIM TARGETS

### METHODS

#### Participants

Twenty-seven infants (14 females, mean age = 4 months; 19 days, range = 3;16–5;12) participated. An additional 25 infants began or completed the procedure but were not included in the final sample due to fussiness (7 infants), procedural error (8), failure to choose either puppet (5), parental interference (2), and general inattentiveness or sleepiness, whereby infants did not attend to puppet events at all (3). The relatively high rate of procedure errors in this condition was due to errors in puppeteering; specifically, even very well trained research assistants occasionally inserted a Helper event when there should have been a Beneficiary event, or a Hinderer event when there should have been a Taker Event. Several studies in the laboratory use box events, and so it was fairly difficult to inhibit the practiced motor repertoires of Helping and Hindering to perform Beneficiary and Victim Events. Because these errors disrupted the meaning of the puppet shows entirely, even one required that an infant be excluded from the sample. 19 of 27 infants were first born.

#### Procedures

Procedures were very similar to those in the Antisocial Target condition in Experiment 1, and counterbalancing was the same. However, instead of the Cow continuously trying and failing to open the box and being alternately helped and hindered by the Pigs, the Pigs took turns trying and failing to open the box and the Cow alternately helped one Pig (the Beneficiary) and hindered the other (the Victim).

***Stimuli phase 1: box beneficiary and victim events.*** All movement details in Experiment 2 were as in Experiment 1, aside from those changes that were necessary to flip the puppets’ agent/patient roles. Each event began when the curtain rose to reveal the Pigs resting at each rear corner of the puppet stage and the box containing a colorful toy in the middle. The Cow then entered from underneath the back curtain, but instead of moving forward and attempting to open the box himself he simply paused just in front of the curtain while one of the Pigs made a failed attempt to open the box. Specifically, during *Beneficiary Events*, the Beneficiary Pig ran forward and looked into the box, and then tried but failed to open it. On the Beneficiary’s fifth failed attempt, the Cow intervened by running around from behind the box to the side of the stage opposite the Beneficiary Pig, and grasped the lid and opened it together with the Beneficiary. The Beneficiary then jumped into the box and lay down on the toy inside, achieving its goal, and the Cow jumped off the box and ran offstage. During *Victim Events*, the Victim Pig ran forward and tried but failed to open the box; on the Victim’s fifth attempt the Cow ran to the side of the box opposite the Victim Pig and jumped sideways onto the box lid, slamming it shut. The Victim jumped off the box and lay his head on the table, failing to achieve his goal, and the Cow jumped off the box and ran offstage. Infants’ attention following each event was coded as in Experiment 1. Once infants’ reached the habituation criterion or watched 14 total Beneficiary and Victim events, the Victim was made the Target of giving and taking during Stimuli Phase 2.

***Stimuli Phase 2: giving and taking events.*** As in Experiment 1, Giving and Taking in Stage 2 was puppeteered by the coder from Stage 1. Giving and Taking Events in Phase 2 of Experiment 2 were absolutely identical to Giving and Taking Events in Phase 2 of Experiment 1. Rather than the Target of Giving and Taking being either the Prosocial or the Antisocial Pig, it was always the Victim Pig.

***Choice phase.*** As in Experiment 1, the choice was presented by the coder from Phase 2 who had puppeteered the Beneficiary/Victim Events during Phase 1. Although Experiment 2 was not “double blind” in the same way as Experiment 1 (because only the Victim condition was run), the choice presenter was entirely unaware of which puppet had been the Giver and the Taker during Phase 2 and so s/he could not unduly influence infants’ choices. An additional 25% of infants’ choices in each condition were recoded for reliability purposes; reliability was 100%.

### RESULTS

Results in the Victim Target are first presented alone, and then compared to infants in Experiment 1.

#### Attention during Stimuli Phase 1

***Rate of habituation.*** Infants in the Victim Target condition habituated in 9.26 (SEM = 0.56; 95% CI [8.11, 9.41]) trials, with 23/27 habituating within 14 trials. The number of habituation events viewed by infants in each condition did not differ [univariate ANOVA on how many events infants viewed during habituation with condition (Prosocial Target, Antisocial Target, Victim Target) as a between-subjects factor: *F*_2,79_ = 2.01, *p* = 0.14; $\eta_{p}^{2}$ = 0.05].

***Attention to beneficiary versus victim events.*** Infants looked equally to the first Beneficiary event and the first Victim event they saw [Beneficiary (SEM) = 8.74 s (1.55); Victim (SEM) = 7.69 s (1.13); paired-*t*_26_ = 0.82, *p* = 0.42, *d* = 0.15; 95% CI [-3.67, 1.57]], and equally to first three Beneficiary and first three Victim events [average three Beneficiary (SEM) = 8.37 s (1.12); average three Victim (SEM) = 8.40 s (1.01); paired *t*_26_ = -0.04, *p* = 0.97, *d* = 0.01; 95% CI [-1.55, 1.63]]. Neither measure of attention to habitation events differed with that of infants in Experiment 1 who viewed Prosocial and Antisocial events (repeated-measures ANOVAs on attention with condition (Prosocial Target, Antisocial Target, Victim Target) as a between-subjects factor revealed neither main effects nor interactions with condition; *F*_2,79_’s < 1.35, *p*’s > 0.24, $\eta_{p}^{2}$ ’s < 0.02). This is not surprising, given all infants viewed the essentially the same alternating Prosocial and Antisocial acts in all three conditions: box shows during Phase 1 only differed based on whether there were two actors and one recipient (in Experiment 1) or one actor and two recipients (in Experiment 2).

***Attention to giving versus taking events during phase 2.*** Infants looked for an average of 6.90 s (SEM = 0.96) to the Giving event and an average of 8.06 s (1.45) to the Taking event during Phase 2; attention did not differ by event type (paired *t*_26_ = -0.86, *p* = 0.40, *d* = 0.18, 95% CI [-1.63, 3.95]). Attention to Giving and Taking events did not differ with either condition in Experiment 1 (repeated-measures ANOVA with condition as a between-subjects factor, *F*_2__,__79_ = 0.01, *p* = 0.99, $\eta_{p}^{2}$ = 0.00).

#### Choice

***Preliminary analyses.*** Preliminary binomial tests revealed no effects of side or color of the Giving Tiger on infants’ choices for Givers over Takers (*p*’s > 0.21), habituators’ choice patterns did not differ from non-habituators’ (Fisher’s Exact *p* = 0.58), boys and girls preferred Givers and Takers at equal rates (Fisher’s Exact *p* = 1.0) and the choice pattern of infants with siblings did not differ from those without (Fisher’s Exact *p* = 1.0). An ANOVA on infants’ choice of the Giver versus the Taker with age as a covariate revealed no effect of age on infants’ choices (*F*_1,26_ = 2.35; *p* = 0.14; $\eta_{p}^{2}$ = 0.09). An additional 25% of infants’ choices were recoded for reliability purposes; reliability was 100%.

***Choice of givers versus takers.*** More infants in the Victim Target condition chose the Giver than chose the Taker (18 of 27 infants; binomial *p* = 0.12; 95% CI contains 50 [48, 81]). Critically, although the rate of choosing the Giver over the Taker did not reach significance in this sample, the rate of choosing the Taker was significantly different in the Antisocial Target and Victim Target conditions [Pearson’s χ^2^ (df = 1) = 10.8, *p* = 0.001]; infants were 44% more likely to choose the Taker in the Antisocial Target condition than in the Victim Target condition (95% CI does not contain 0 [18, 63]). This result suggests that infants in the Antisocial Target condition in Experiment 1 did not choose the Taker based on valence-matching alone. That said, the rate of choosing the Taker was *also* significantly different between the Prosocial Target and Victim Target conditions [Pearson’s χ^2^ (df = 1) = 4.12, *p* = 0.04]; infants were 23% less likely to choose the Taker in the Prosocial Target condition the Victim Target condition (95% CI contains 0 [0, 43]). Implications for this result will be addressed in the discussion.

## GENERAL DISCUSSION

Results from the current studies suggest that given sufficient time to process prosocial and antisocial events, 4.5-month-olds are capable of evaluating others’ actions in context. In contrast to past work in which younger infants preferred prosocial Givers over antisocial Takers regardless of the past actions of the Target of those behaviors, when 4.5-month-olds were habituated to the past prosocial or antisocial actions of a Target they preferred Givers to Prosocial Targets and Takers from Antisocial Targets. That younger infants require more time to process and/or remember events than do older ones has been consistently demonstrated in developmental psychology research (see Roveé-Collier, 1997, 1999; Hayne, 2004; Colombo and Mitchell, 2009), the current studies demonstrate that similar information processing and memory limitations may underlie early failures to demonstrate context-dependent social evaluation. Notably, infants in the current studies were only habituated to prosocial and antisocial box events (not to giving and taking ball events): they were shown only one giving and one taking act before asked to choose between the Giver and the Taker. This indicates that 4.5-month-olds can evaluate others’ actions in context on their *very first* observations of valenced actions directed toward a valenced target, so long as their representation of that target is sufficiently strong.

The current studies used preferential reaching as a measure of infants’ social evaluations. Experimental methodologies are necessarily limited to those behaviors infants are physically capable of performing; indeed, the current studies lowered the age at which we achieved successful reaches from infants under 4.5 months of age. Though there are presumably countless reasons why infants would touch one puppet versus another; including social evaluation but also including perceptual interest, confusion or curiosity, absence of fear, etc., other work using the very same box events as in the current Stimuli Phase 1 has revealed that 16-month-olds selectively match the food preferences of prosocial puppets but not antisocial ones, and 21-month-olds selectively give resources to prosocial puppets and take them from antisocial ones (Hamlin et al., 2011; Hamlin and Wynn, 2012). Thus, although it is critical to continue assessing at what level young infants’ reaching behaviors reflect true “evaluation,” this developmental continuity suggests it is appropriate to (cautiously) do so.

Infants’ context-dependent evaluation did not stem entirely from simple valence-matching mechanisms: when infants viewed a Giver and a Taker act on a Victim Target, more preferred the Giver, a significantly different pattern of choice than infants in the Antisocial Target condition. Intriguingly, infants were *also* significantly more likely to choose the Giver in the Prosocial Target than in the Victim Target condition: 89% of infants chose a Giver to a Prosocial Target, whereas 67% of infants chose a Giver to a Victim Target. This pattern was not observed in the corresponding conditions Hamlin et al. (2011), wherein 8- and 19-month-olds preferred Givers to Prosocial Targets and Victim Targets at the same rate; furthermore, here fully 2/3 of infants preferred Givers to Victims. Despite this, if the difference in rate of choosing Givers to Prosocial versus Victim Targets were to replicate it would be consistent with several potential explanations. First, perhaps valence-matching mechanisms play a role in early social evaluations but are not entirely responsible for them. For instance, perhaps 4.5-month-olds *do* like those whose interactions serve to maintain action valence through time, as some form of valence-based familiarity preference, thereby weakening their preference for Givers to Victims. That the same asymmetry was not observed in 8- or 19-month-olds in Hamlin et al. (2011) suggests either that the asymmetry reflects early confusion about the roles of agent versus patient in social interactions that is overcome by 8 months of age, or that associative effects are relatively weak and emerge only after someone has been victimized repeatedly, as in the current habituation methodology. Alternatively, the asymmetry in preference for Givers to Prosocial versus Victim Targets could reflect some tendency to “blame the victim,” as has been demonstrated in adults and young children (Piaget, 1932/1965; Lerner, 1980; Jost and Hunyady, 2002; Olson et al., 2006; Olson et al., 2008). Indeed, children show preferences for lucky and against unlucky individuals even when the lucky and unlucky events were clearly random (finding 5$, getting caught in the rain); in the current studies, given that the Cow engages in *both* prosocial and antisocial acts and directs antisocial acts toward the Victim specifically, infants may have had reason to suspect that the Victim’s lot was non-random. Future work might examine whether infants show a tendency to “blame the victim” (defined by smaller preferences for Givers to Victims than for Givers to Prosocial others) in a condition in which Beneficiaries are helped and Victims are hindered by two distinct individuals, as this might provide less reason to view Beneficiaries and Victims as deserving their treatment.

Herein, the general terms “context-dependent” and “global” social evaluation have referred to a very specific form of context-based evaluation, asking whether infants prefer those who intentionally harm those who have intentionally harmed others. Above and beyond the very simple valence-matching mechanisms that do not account (entirely) for infants’ preferences, there remain several possibilities for the exact nature of infants’ global evaluations, not disentangled by the present work. For instance, past work suggests that infants utilize and privilege intention in their assessment of others for their third-party prosocial and antisocial acts by 8–10 months of age (Hamlin, 2013b; Hamlin et al., 2013b). Do mental states influence context-dependent evaluations? If so, whose mental states matter? For instance, would infants prefer those who try but fail to harm intentional hinderers to those who try but fail to help them? Would they prefer those who intentionally harm someone who only accidentally harmed someone else? In a different form of context-specific evaluation, it is as of yet unclear to what extent the *relationship* between an agent and a patient influences infants’ evaluations of intentional helping and hindering acts. For instance, if infants could identify a hindering agent as a “caregiver” of another agent, would they evaluate the hinderer differently? Adults regularly excuse (or applaud) caregivers’ hindering acts, based on the assumption that the target needs protection rather than deserves mistreatment; it is an open question whether infants do the same.

Although the current results suggest that some form of domain-general development is responsible for previously observed developmental differences in context-dependent social evaluation, it remains unclear exactly what domain-general process is responsible, or whether it is a combination of several interrelated processes. Did 5-month-olds in Hamlin et al. (2011) fail to *encode* the prosocial and antisocial acts in Stimuli Phase 1? Or was it that they failed to *remember* who did what over the (very) brief delay during which the second puppet show was set up? Or was it the context-change from box to ball events that disrupted infants’ *retrieval* of the puppets’ past actions? Although young infants have some difficulty with each of these processes (Hayne, 2004), an encoding failure seems the least likely: infants succeeded at distinguishing Givers and Takers after observing only one of each behavior in Stimuli Phase 2, both here and in past work. Thus, it does not appear that 8-month-olds are simply more efficient processors of prosocial and antisocial actions in general. However, simple time-decayed forgetting versus context-disrupted retrieval are currently difficult to tease apart. Although there is a delay after Stimuli Phase 2 during setup for the Choice during which infants do not forget who did what in Stimuli Phase 2 (if they did they should not be able to distinguish Givers and Takers at all), the delay after Stimuli Phase 1 is longer, as it requires trading experimenters, exchanging stimuli, reading scripts to identify which puppets are involved, etc. If infants’ memory for who did what is already weakened at the start of Phase 2, then the change in context with the introduction of a new puppet show might wash it away entirely. Future study might examine whether utilizing more-similar contexts across Phases would improve younger infants’ performance.

Furthermore, these results raise the question of exactly what it is that infants in the current studies *habituate to*: upon habituation, what is contained in infants’ internal representations of prosocial and antisocial box events? A representation of the acts themselves and their valence? A representation of the relationship between the helper and hinderer and the target of their actions? Or perhaps a representation of the evaluation of the helper’s and hinderer’s traits? Work with adults would suggest the latter (e.g., Todorov and Uleman, 2003); but young children do not readily predict future from past behaviors, presumably due to a failure to attribute traits (e.g. Boseovski and Lee, 2006). Future studies might explore these questions by examining infants’ expectations for the future behaviors of helpers and hinderers. For instance, do infants see former helpers and hinderers as more likely to perform the same action again toward a new individual, to perform a similar action in such a way that it is no longer valenced, or to perform a different kind of action that is of the same valence?

Finally, demonstrating that 4.5-month-olds *can* evaluate others in context given sufficiently supportive methodological design should not be taken as evidence that they routinely do so in their daily lives. First, given their first-born status and very young age, it is relatively unlikely that our infants regularly (if ever) observe overtly antisocial behaviors directed at individuals whom they have also observed behaving antisocially; at least of the kind demonstrated here that involve neither valenced facial expressions nor linguistic cues. Even if such behaviors were to occur in infants’ environments, everyday social observations presumably do not provide sufficient information to *habituate* infants to others’ prosocial and antisocial acts, and the current studies demonstrate that habituation is required for young infants to demonstrate context-dependent social evaluation. Although this could be viewed as a limitation of this work, the present findings nonetheless suggest that given sufficient support, young infants’ social evaluations share important commonalities with older infants’ and children’s, informing our understanding of the nature and developmental progression of social evaluation in infancy.

## Conflict of Interest Statement

The author declares that the research was conducted in the absence of any commercial or financial relationships that could be construed as a potential conflict of interest.
